# Supplementary material for: Convergent Genomic Signatures of High-Altitude Adaptation among Six Independently Evolved Mammals
Source: Animals (Basel). 2022 Dec 16;12(24):3572. doi: 10.3390/ani12243572 (PMC9774524; doi:10.3390/ani12243572)
Supplement: Supplementary file 1 [file animals-12-03572-s001.zip › Supplementary Figures.pdf]

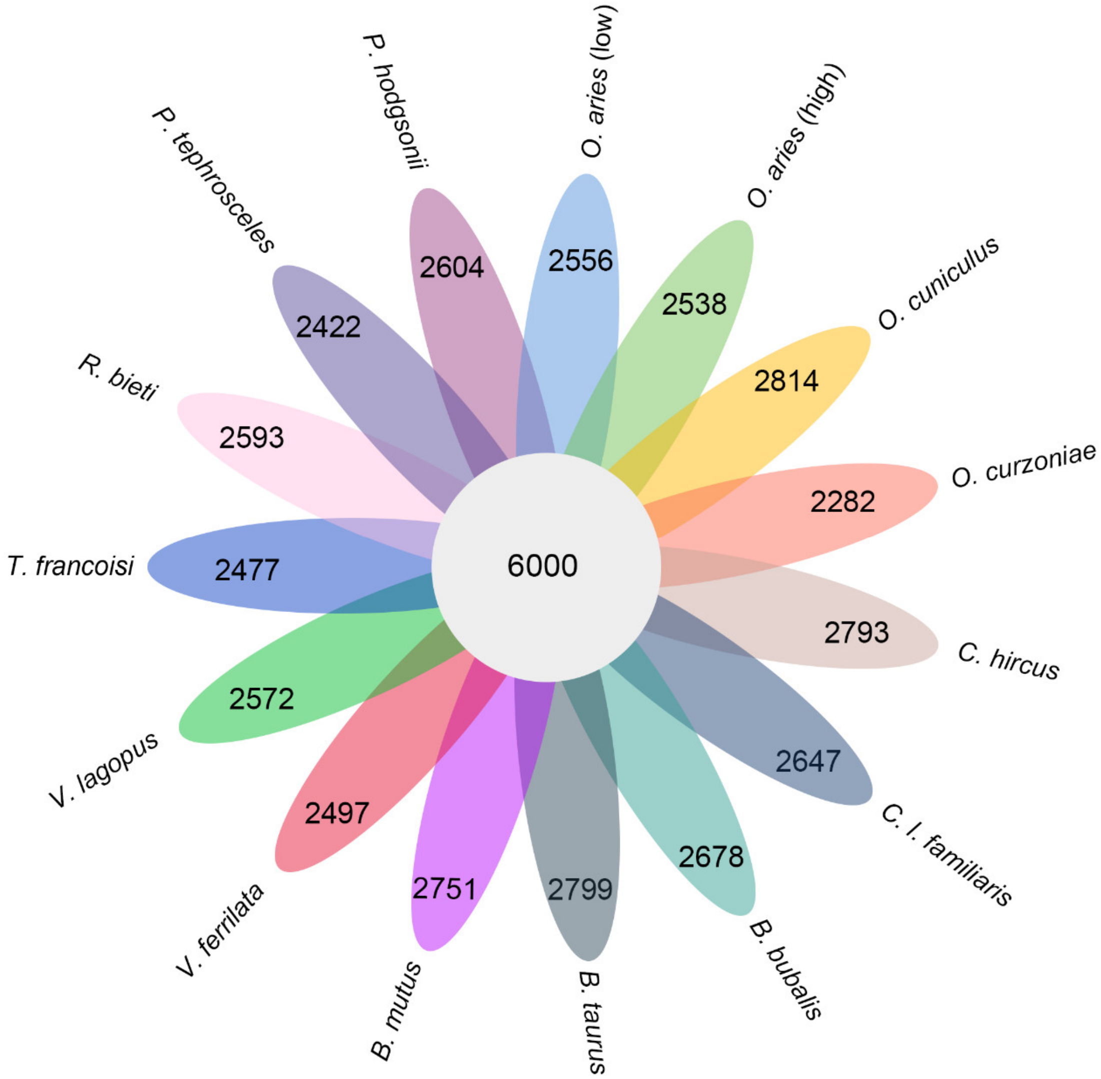

Figure S1. Statistics of the number of single copy genes of 15 species. The middle of the petal represents the one-to-one single copy homolog genes shared by 15 species.

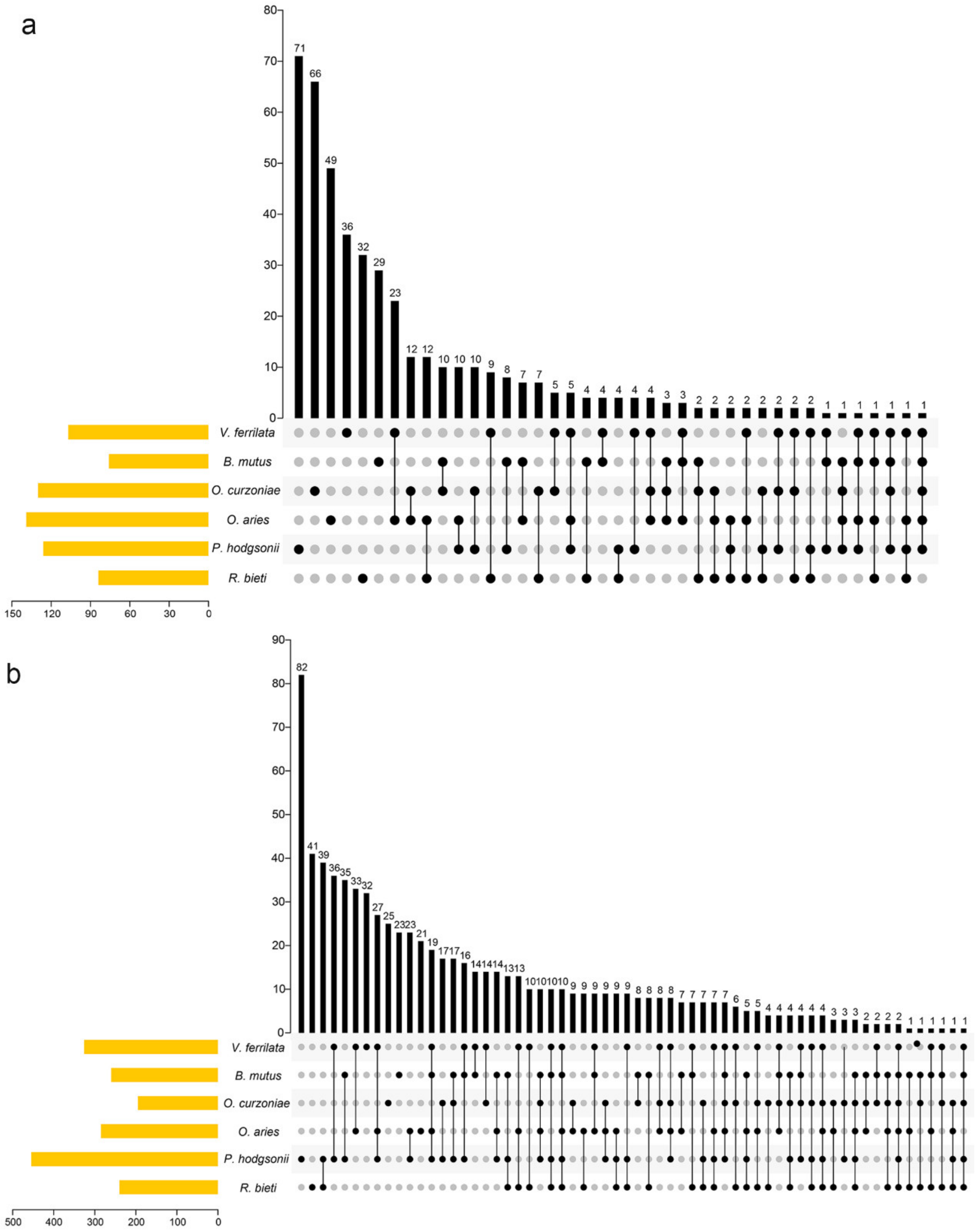

Figure S2. Upset statistical analysis of categories of significantly altered gene families in six high-altitude species. The black dots represent gene families that are significantly altered in the species, and the gray dots represent gene families that are not significantly altered in the species. Each bar represents the number of gene families in that category. (**a**) Upset statistical analysis of categories of significantly expanded gene families in six high-altitude species. (**b**) Upset statistical analysis of categories of significantly contracted gene families in six high-altitude species.
