# Supplementary material for: Convergent Genomic Signatures of High-Altitude Adaptation among Six Independently Evolved Mammals
Source: Animals (Basel). 2022 Dec 16;12(24):3572. doi: 10.3390/ani12243572 (PMC9774524; doi:10.3390/ani12243572)
Supplement: Supplementary file 1 [file animals-12-03572-s001.zip › Supplementary Tables.pdf]

Table S1. Genome accession number and gene number used for gene family clustering in each species.

Species	NCBI accession number	Unclustered	Single copy	Multiple copy	Others	Total
Bos mutus	GCF_000298355.1	282	8751	2246	8325	25630
Bos taurus	GCF_002263795.1	422	8799	2193	9240	26700
Bubalus bubalis	GCF_019923935.1	508	8678	2552	9193	26986
Canis lupus familiaris	GCF_014441545.1	733	8647	2555	8526	26537
Capra hircus	GCF_001704415.1	287	8793	2219	8816	26148
Ochotona curzoniae	GCF_017591425.1	282	8282	3405	8352	26733
Oryctolagus cuniculus	GCF_000003625.3	731	8814	2100	6349	24265
Ovis aries (high)	GCA_017524585.1	1403	8538	2834	7506	26485
Ovis aries (low)	GCF_016772045.1	338	8556	2918	9011	26860
Pantholops hodgsonii	GCF_000400835.1	450	8604	2646	8373	30828
Piliocolobus tephrosceles	GCF_002776525.3	1293	8422	3178	8848	29378
Rhinopithecus bieti	GCF_001698545.1	867	8593	2636	8142	26301
Trachypithecus francoisi	GCF_009764315.1	623	8477	3255	8659	27098
Vulpes lagopus	GCF_018345385.1	696	8572	2842	8214	26353
Vulpes ferrilata	GCA_024500485.1	2653	8497	3073	6907	27369

Table S2. The number of significantly expanded gene families enriched in different GO terms of each species.

GO term	*B. mutus*	*P. hodgsonii*	*O. aries*	*V. ferrilata*	*R. bieti*	*O. curzoniae*
GO:0003676	7	1	37	30	12	15
GO:0003735	12	117	64	37	9	9
GO:0004930	309	26	124	4	38	230
GO:0005509	27	17	3	15	14	34
GO:0005515	3	9	35	51	26	67
GO:0005576	18	12	26	20	3	21
GO:0005634	4	4	32	23	11	23
GO:0005840	12	111	64	37	9	9
GO:0006355	7	13	8	15	57	51
GO:0006412	12	117	64	37	9	9
GO:0007186	324	39	135	4	42	264
GO:0016020	27	26	19	13	26	40
GO:0016021	327	45	169	10	43	264
GO:0046982	44	17	12	8	2	61
GO:0003677	44	31	19	12	3	80
GO:0007156	25	12	3	12	11	34
GO:0000786	27	17	0	1	2	61
GO:0003723	13	0	35	43	2	12
GO:0003924	5	34	19	25	0	14
GO:0004984	321	28	128	0	382	205
GO:0005198	1	0	12	10	3	3
GO:0005525	19	37	52	37	0	14
GO:0006457	2	12	9	3	0	3
GO:0006508	4	7	1	0	11	32
GO:0008270	1	8	6	0	18	14
GO:0003824	0	66	8	47	3	4
GO:0005524	0	21	29	30	2	22
GO:0006749	0	1	4	17	4	15
GO:0016887	0	7	5	30	2	12

Table S3. The number of significantly contracted gene families enriched in different GO terms of each species.

	B. Mutus	*P. hodgsonii*	*O. aries*	*V. ferrilata*	*R. bieti*	*O. curzoniae*
GO:0004984	130	239	117	27	16	26
GO:0005525	6	13	8	10	9	1
GO:0005515	6	11	12	1	8	3
GO:0020037	5	16	12	3	7	1
GO:0005634	7	3	5	8	5	2
GO:0004930	139	236	104	19	15	26
GO:0006355	23	8	22	26	33	7
GO:0003676	11	8	1	6	4	7
GO:0016021	151	249	129	32	20	39
GO:0005524	9	14	2	6	15	1
GO:0007186	149	244	125	27	16	39
GO:0003735	0	9	33	40	62	26
GO:0006123	1	0	2	2	1	1
GO:0005840	0	9	33	38	54	25
GO:0006412	0	9	33	40	62	26
GO:0005126	24	12	6	0	5	2
GO:0006952	24	12	6	0	5	2
GO:0005200	6	7	7	8	4	0
GO:0016459	9	6	2	5	9	0
GO:0051015	9	6	2	5	7	0
GO:0007017	6	7	8	8	4	0
GO:0016491	2	0	14	3	1	6
GO:0004252	2	2	2	1	2	0
GO:0005751	1	0	2	2	1	1
GO:0000786	22	26	18	29	34	0
GO:0003774	9	6	2	5	9	0
GO:0003677	24	31	19	29	35	0
GO:0005874	6	7	7	8	4	0
GO:0045095	5	20	7	7	4	0
GO:0005576	26	17	20	0	5	3
GO:0003723	9	12	0	5	5	8
GO:0003824	0	4	4	1	1	4
GO:0005506	1	12	7	1	7	0
GO:0005739	1	1	1	2	3	0
GO:0046982	23	32	19	29	36	0
GO:0003924	6	7	8	10	9	0
GO:0006508	4	2	2	1	2	0
